# One-Week Dynamic Changes in Cardiac Proteomes After Cardiac Radioablation in Experimental Rat Model

**DOI:** 10.3389/fcvm.2022.898222

**Published:** 2022-06-28

**Authors:** Byoung Hyuck Kim, Jin Woo Jung, Dohyun Han, Myung-Jin Cha, Ji Hyun Chang

**Affiliations:** ^1^Department of Radiation Oncology, Seoul Metropolitan Government Seoul National University Boramae Medical Center, Seoul, South Korea; ^2^Department of Radiation Oncology, Seoul National University College of Medicine, Seoul, South Korea; ^3^Proteomics Core Facility, Transdisciplinary Research and Collaboration, Biomedical Research Institute, Seoul National University Hospital, Seoul, South Korea; ^4^Transdisciplinary Department of Medicine and Advanced Technology, Seoul National University Hospital, Seoul, South Korea; ^5^Division of Cardiology, Department of Internal Medicine, Asan Medical Center, University of Ulsan College of Medicine, Seoul, South Korea

**Keywords:** radioablation, cardiac conduction system, proteomics, rats (all MESH terms), antiarrhythmic effects

## Abstract

**Background:**

Recently, stereotactic ablative radiotherapy (SABR) has been adopted to non-invasively treat catheter ablation-refractory ventricular tachycardia (VT). VT episodes have been dramatically reduced after SABR, within weeks; however the underlying mechanisms of these clinical effects and potential mediators of early anti-arrhythmic effect remain unclear.

**Methods:**

In this study, cardiac tissue was harvested from non-irradiated control (0 Gy), conventional irradiated control (2 Gy), and radioablative test (25 Gy) rat groups after 3 and 7 days of irradiation. The samples were proteomically analyzed to identify the differentially expressed proteins (DEP) between different groups. Validation experiments were performed similar to validation in profiling where Data independent acquisition and parallel reaction monitoring methods were used. Data are available *via* ProteomeXchange with identifier PXD030878.

**Results:**

Functional enrichment analysis of 25 Gy sample showed that among the downregulated proteins, “intracellular signal transduction” and “cell to cell adhesion” proteins were significantly affected at day 3 while “Ras protein signal transduction,” “GTPase regulation,” and “actin filament-based process” proteins were majorly affected at day 7. GO analysis demonstrated that most of the upregulated proteins belonged to the classes “cellular stress response,” “endomembranal organization,” or “endoplasmic reticulum stress response” at day 3. At day 7, 42 proteins, mainly associated with response to drug, organic substance, or radiation, were specifically upregulated in 25 Gy. DEP analysis of cardiac conduction showed Ryr2 and Cav1 upregulation and Cacna2d2, Gja3, Scnb2, and Kcnn3 downregulation in the 25 Gy group compared to 0 Gy. In validation experiments, four proteins (Gsta1, Myot, Ephx1, and Capg) were repeatedly detected with 25 Gy-specific patterns at day 7.

**Conclusions:**

25 Gy single fractional irradiation induces considerable cardiac proteome changes within the first 7 days, distinct from 2 Gy. Several candidate proteins displayed 25 Gy-specific changes and were related to oxidative stress-induced innate response or cardiac remodeling processes. Future studies should explore the specific role of these proteins upon cardiac radioablation.

## Introduction

Radiation therapy (RT) has been used for several non-malignant diseases such as degenerative arthritis or inflammatory diseases (1). Recently, cardiac radioablation using stereotactic ablative radiotherapy (SABR)/stereotactic body radiation therapy (SBRT) has been adopted to treat catheter ablation-refractory ventricular tachycardia (VT) by delivering high-dose radiation to VT focus non-invasively (2). Although VT episodes are reported to dramatically decline after cardiac radioablation within days to few weeks (3), the underlying mechanism of these clinical effects remains unknown.

It has been reported that ablative RT in tumors proceeds *via* a short-term apoptotic cell death, vascular injury, and long-term fibrosis or tissue necrosis (4). However, limited information is available about the cellular injury mechanisms specific to non-malignant arrhythmogenic cardiac tissues. Clinically observed early electrical changes within few weeks cannot be explained by RT-induced fibrosis that emerges in several months or years. Previous pre-clinical studies did report RT-induced late cardiac fibrosis that blocked conduction, but could not explain how single high-dose RT reduced VT burden rapidly, before the development of fibrosis (5, 6).

Recent transcriptomic analysis identified notch signaling as a possible contributor to conduction reprogramming after cardiac radioablation, in the absence of fibrosis (7). However, to date, no proteomic strategies have identified biological changes of cardiac radioablation. Proteomics investigates large-scale gene expression linked to the protein levels and is closely related to the disease phenotype compared to the genome. Therefore, it can identify biomarkers explaining cardiac radioablation-induced early antiarrhythmic effects and help in understanding the molecular basis of this novel treatment technique.

In this study, irradiated rat hearts were proteomically analyzed to identify differentially expressed proteins between different groups and to describe biological changes caused by single high-dose irradiation.

## Methods

### Experimental Design

All animal experiments were approved by the Institutional Animal Care and Use Committee at the Seoul National University Hospital (IACUC No. 18-0245-S1A0). Male wild type Lewis rats aged 9–10 weeks were used for this study. Rats were housed at room temperature under a 12-h light-dark cycle with access to water and chow *ad libitum*. We divided experimental rats randomly into three groups as follows: non-irradiated control 0 Gy, conventional irradiated control 2 Gy, and radioablative group 25 Gy (radiation dose used in previous clinical studies) because conventional fractional dose 2 Gy might differ in terms of conduction modulation properties compared to 25 Gy. After anesthesia using isoflurane, fixed rats in customized acryl zigs were irradiated each assigned dose using the megavoltage linear accelerator (ClinacIX, Varian). The RT field was set to sufficiently cover whole heart and other areas were blocked by a multileaf collimator.

### Sample Preparation

Harvesting of cardiac tissue was performed 3 and 7 days after irradiation. Under anesthesia, the whole heart was extracted through routine thoracotomy and about 100 mg of ventricular apex was cut and immediately frozen in liquid nitrogen. Heart tissue samples were prepared using a filter-aided sample preparation (FASP) modified method for frozen tissue preparation (8). Frozen tissue samples were homogenized with lysis buffer (4% SDS, 2 mM tris (2-carboxyethyl)phosphine, and 0.1 M Tris-HCl, pH 7.4) and the protein concentration was determined by tryptophan fluorescence emission at 350 nm and an excitation wavelength of 295 nm. Proteins were digested using the 2-step FASP procedure with some modifications. The digested peptides were acidified using 10% trifluoroacetic acid and desalted using homemade C18-StageTips as previously described (8, 9). Finally, we used a vacuum dryer to dry and store the sample at −80°C.

### TMT 10-Plex Labeling

Tandem mass tag (TMT) labeling was performed according to the manufacturer's protocol with some modifications (10). The TMT-labeled peptides were pooled at a 1:1:1:1:1:1:1:1:1:1 ratio, and the mixtures were dried in a speed vacuum.

### LC-MS/MS Analysis

All TMT 10-plex samples and Data independent acquisition (DIA) quantification samples were analyzed by LC–MS/MS using quadrupole orbitrap mass spectrometers, Q-Exactive HF-X (Thermo Fisher Scientific, Waltham, MA, USA) coupled with an Ultimate 3000 RSLC system (Dionex, Sunnyvale, CA, USA) consisting of EASY-Spray^™^ LC columns with an electrospray source, and the column temperature of 60°C. For spectral library construction, the mass spectrometer was operated in data-dependent acquisition mode. The survey MS scan (350–1650 m/z) was acquired at a mass resolution of 70,000 at m/z 200, and the MS/MS spectrum was acquired at a mass resolution of 17,500 at m/z 200.

### Data Processing for TMT Data

MS raw files were processed using Proteome Discoverer version 2.4 with the SEQUEST-HT algorithm against the Rat UniProt reference database (September 2018, 37,316 entries).

### Data Processing for DIA Data

The DIA data of individual samples was analyzed with Spectronaut Pulsar version 14 (Biognosys). We converted the DIA raw files into an htrm format using the GTRMS converter provided by the Spectronaut software. The FDR was estimated with the mProphet (11) approach and set to 1% at peptide precursor level and at 1% for protein level. The quantification information was acquired at the protein level by using the q-value <0.01 criteria, and used for subsequent analyses.

### Parallel Reaction Monitoring Analysis

After proteome profiling analysis with tandem MS spectrometry, we validated the results using parallel reaction monitoring (PRM). Before analysis, we prepared the stable isotope standards (SIS) peptides containing exact equivalent amino acid sequences with unique peptides of target proteins. Those were synthesized with heavy labeled isotope of carbon and nitrogen (13C and 15N) on arginine or lysine (JPT peptide Technologies GmbH, Berlin, Germany). Sequences were selected based on SRMAtlas database (http://www.srmatlas.org) that contains archived transition data from quadrupole-orbitrap MS and from in-house rat heart spectral library. We selected at least three unique peptides per each target protein. The 250 fmol of SIS peptides were spiked into each sample and the peak abundance ratio between SIS peptides and target peptides were used for quantification comparison. The acquired MS data such as peak area integration, ratios, coefficient variance (CV), and retention times were manually adjusted within Skyline software (12). The statistical analysis that compared ratios between time interval and conditions was performed with MSstats (v3.13.7) (13), embedded in Skyline.

### Statistical Analyses

Perseus software was used for all statistical analyses of TMT data (14). Reporter ion intensities were log2-transformed. After the data was normalized using width adjustment in Perseus software, analysis of variance (ANOVA) or two-sample *t*-tests were performed using permutation-based FDR and a significance level of 5%. For clustering analysis, normalized protein abundance levels were subjected to further z-normalization followed by hierarchical clustering in terms of the Euclidean distance and average linkage. After profiling analysis, we verified selected DEPs by DIA analysis. The statistical analysis was also performed with Perseus software. Protein intensities obtained from DIA analysis were log_2_ transformed for further manipulation and filtered proteins which were detected in less than 70% of the total samples. Reverse and contaminant detections were subsequently removed. Missing values were replaced using the normal distribution imputation method with default settings to develop reliable differentially expressed proteins (DEPs). Those filtered proteins were normalized by width adjustment that subtracts the second quartile value (q2) from each value and center the distribution in an asymmetric manner. After normalization, we performed multiple sample test; the ANOVA test and student *t*-test for protein expression changes. The proteins with significant expression changes were determined with *P*-value <0.05 and fold change (FC) lower than 0.84 or >1.2 (*Log*_2_ *FC* >0.26303 or *Log*_2_*FC* <-0.26303).

### Bioinformatics Analysis

To investigate the functional features of DEPs from the ANOVA and student *t*-test, gene ontology (GO) analysis was implemented to determine molecular function or biological process by using DAVID tool (version 6.8) (15). Among the results from functional analysis, we categorized specific GO terms data based on protein expression patterns and filtered by *P*-value lower than 0.05 and matched to cardiac activity and ion channel transfer activity. The PPI (protein-protein interaction) network analysis was performed with Cytoscape software (version 3.7.2) (16) and interaction database was accessed by STRING (Search Tool for the Retrieval of Interacting Genes) (version 10) (17). The expression alternation of DEPs was displayed as colorimetric scheme of Log_2_ FC values; blue for down-regulated proteins, and red for up-regulated proteins, within Cytoscape tool. For pathway analysis, KEGG pathway analysis was performed within KEGGScape (0.9.1) (18). The exhibited DEPs were arranged by the biological process terms that we selected and presented on PPI network.

## Results

### Single Fractional Irradiation Induces Considerable Cardiac Proteome Changes During the First Week

**Figure 1:**
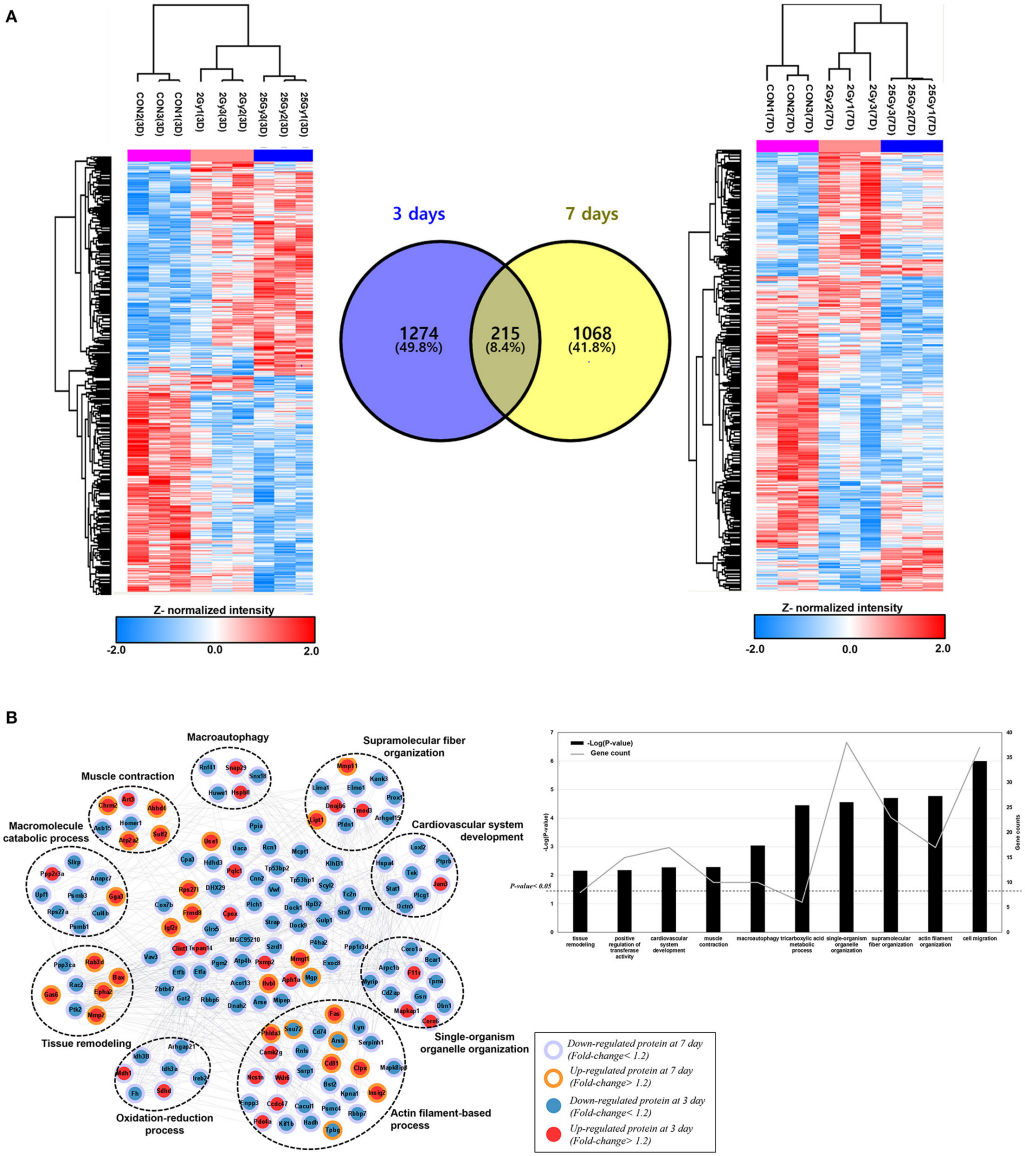
The hierarchical clustering of differentially expressed cardiac proteome induced by single high-dose irradiation. **(A)** Overall ANOVA results at day 3 and day 7. The DEPs were filtered with *P*-value lower than 0.05 and Z-normalized for visualization. The result identified 1,489 and 1,283 DEPs at day 3 and day 7, respectively. The Venn diagram presented the 215 common DEPs. **(B)** PPI (protein-protein interaction) analysis of common DEPs, presented with functional enrichment (*P*-value <0.05) from GO analysis. The filled node presents protein expression from day 3 and the barrier of node presents protein expression from day 7. The list of identified functional process is also displayed as bar chart.

The cardiac proteome of rat was analyzed at days 3 and 7 after 0, 2, and, 25 Gy (*n* = 3, each) single fractional irradiation. The overall scheme of analysis is shown in Supplementary Figure 1. Label-free analysis of the harvested tissue identified 8,484 unique proteins of which 7,938 could be quantified (Supplementary Figure 2). Of these, 1,489 at day 3 and 1,283 at day 7 were found to be differentially expressed proteins (DEPs) with *P*-value lower than 0.05 among the three groups. Figure 1A shows a distinct protein expression pattern at each time point. Coefficient variance distribution, dynamic range of protein abundance, and principle-component analysis plot comparison between irradiation doses are summarized in Supplementary Figure 3.

A total of 215 proteins were significantly differentially expressed at both day 3 and day 7. To discover biological process or molecular function of those, PPI (protein-protein interaction) and GO (gene ontology analysis) were performed with 215 common DEPs. Functional enrichment based on GO analyses demonstrated a possible correlation between single fractional irradiation and several physiological functions (Figure 1B). Gene ontology enrichment analysis for these proteins identified that biological processes: “actin filament-based process” and “single-organism organelle organization” were most affected. Proteins were mainly involved in the actin-filament based process, muscle contraction, oxidation-reduction process, or cardiovascular system development (Figure 1B). These results suggested that single fractional irradiation affected the expression of many cardiac proteins, involved in the contractile function of cardiomyocytes, during first week of exposure.

**Figure 2:**
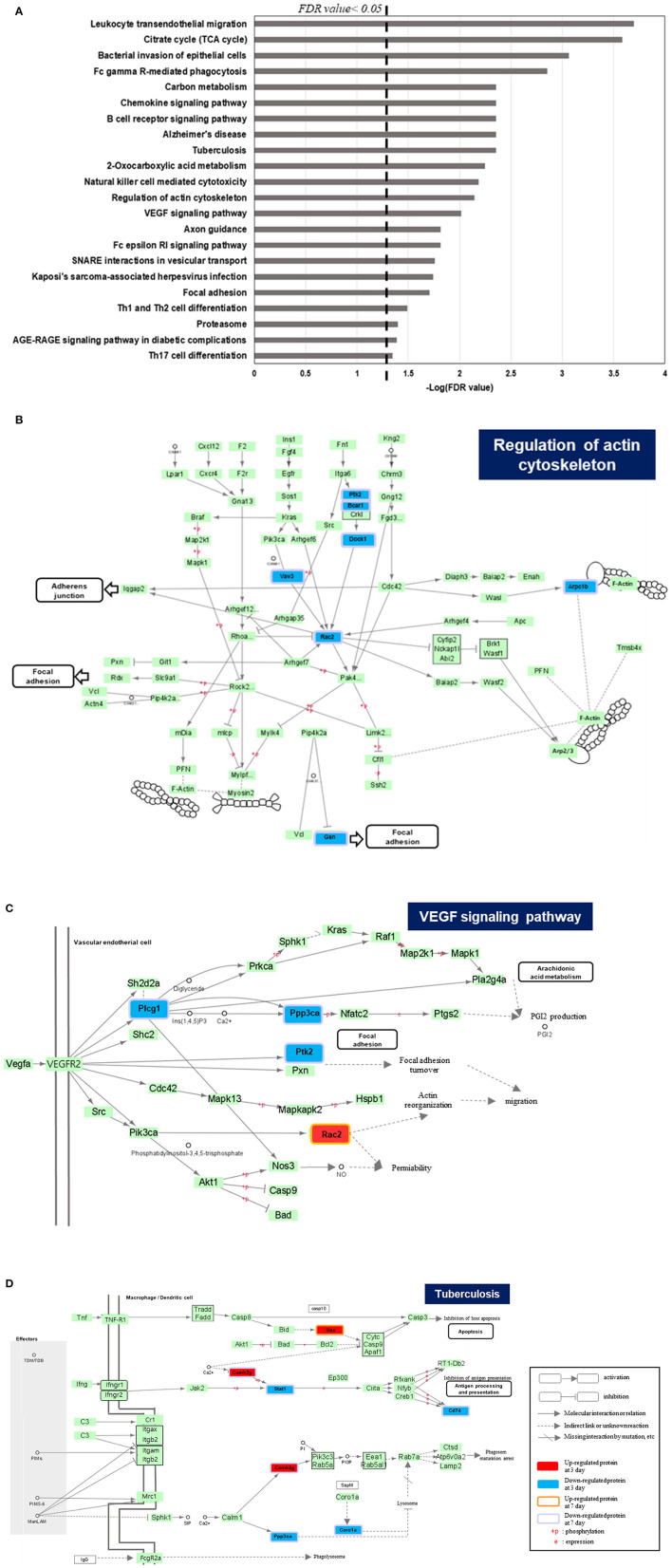
KEGG pathway analysis of common DEPs between 3 and 7 days of dose group in irradiated rat. **(A)** The result of KEGG pathway analysis, filtered with FDR value lower than 0.05. Among those, the pathways that directly related with cardiovascular activity or associated disease are plotted, which are **(B)** Regulation of actin cytoskeleton, **(C)** VEGF signaling pathway, and **(D)** tuberculosis.

Furthermore, those common DEPs were plotted on KEGG pathway analysis (19) to understand the molecular interactions and biological effects on specific network. The analysis was performed with KEGGScape background, embedded in Cytoscape tool (version 3.8.2). The pathway analysis was filtered with FDR value lower than 0.05 and able to obtain 22 statistically significant pathway (Figure 2A). The result indicated that most of identified pathways were equivalent with those from GO analysis, such as regulation of actin cytoskeleton or axon guidance. Among those, immune related pathways; natural killer cell mediated cytotoxicity or T helper cell differentiation, and cell transport related pathways; leukocyte transendothelial migration or vesicular transport pathway, are discovered. Moreover, we investigate the protein expression changes that are related with cardiovascular physiology such as tuberculosis (rno05152), VEGF signaling pathway (rno04370), and regulation of actin cytoskeleton (rno04810). Most of proteins categorized within actin cytoskeleton or VEGF signaling pathway were down-regulated after 25 Gy irradiation from both of day 3 and day 7 (Figures 2B,C). In general, the accumulation of cytoskeletal proteins such as tubulin, desmin, and membrane associated proteins might be the compensatory mechanism of heart malfunction independent of the fundamental cardiac disease (20). As the actin or microtubules role a repairing process and serve to transport membrane and other components to the wound (21), the downregulated proteins in the pathway might reflects the wound healing effects, and able to affect redox regulation (22).

As the high concentration of VEGF (vascular endothelial growth factor) family are observed in various cardiovascular disease and associates poor prognostic (23), the identified DEPs on VEGF related pathway were also explored. The down regulated proteins in VEGF signaling pathway which are Plcg2 (1-phosphatidylinositol 4,5-bisphosphate phosphodiesterase gamma-2), and Ppp3ca (serin/threonine-protein phosphatase 2B catalytic subunit alpha) inhibit PGI2 production and it would lead decrease the VEGF expression (Figure 2C).

Tuberculosis (TB) is a bacterial infection disease that mostly affects lung but, it could attack other parts of organs such as kidney, brain, and heart. Therefore, cardiac involvement in TB is common and TB infected patients possess a high risk to cause heart failure, constrictive pericarditis, or developing atherosclerotic cardiovascular diseases (24, 25). The Cd74 (H-2 class II histocompatibility antigen gamma chain) included in TB pathway is reported that its expression on atherosclerotic plaques, and peripheral blood mononuclear cells (PBMC) with its potential pro-inflammatory responses (26). Likewise, Coro1a (Coroin-1A) that also regulated TB pathway, was studied that the depletion of its expression significantly leads the activation of p38β and it could protects endothelial cells from apoptosis, induced by TNFα activity (27). As those are specifically downregulated proteins after 25 Gy after day 3 and 7, it is expected that better prognosis related with cardiovascular disease (Figure 2D).

### Functional Enrichment Analysis of 25 Gy Sample-Specific Proteins by Hierarchical Clustering

**Figure 3:**
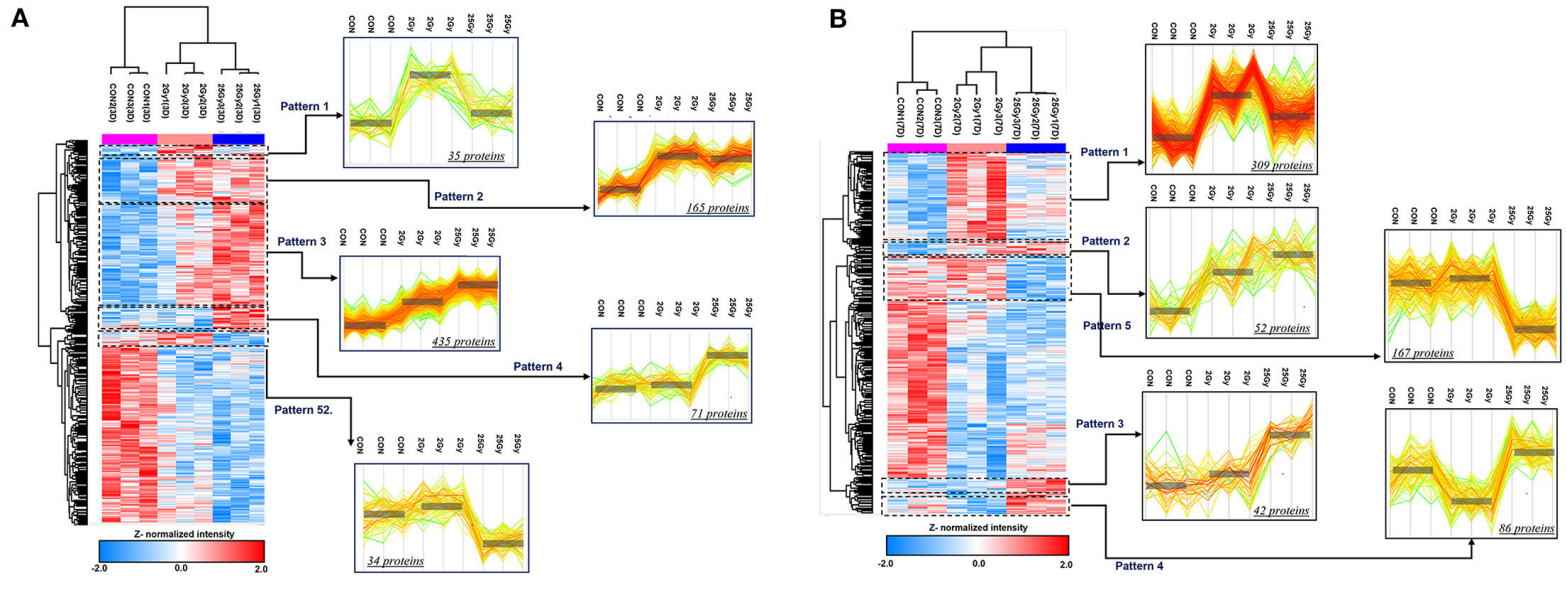
ANOVA pattern clustering analysis of differentially expressed proteins related to dose group in irradiated rat. The hierarchical cluster was performed after ANOVA multiple comparison test between 0, 2, and 25 Gy at day 3 and day 7, respectively. The pre-process was implemented by unbiased K-means clustering. Among the defined clusters, we subdivide those to investigate the detailed pattern of proteome alterations related to dose of irradiation. The colorimetric scheme represented z-normalized intensity of DEPs. **(A)** Defined clusters that reveals specific expression pattern of DEPs at day 3 (*P*-value <0.05, 1,489 proteins). The clusters were divided with 2.245 threshold. The detailed clusters show the protein alteration patterns with specifically up-regulated in 2 Gy irradiation (pattern 1), gradually increasing according to increasing dose of irradiation (pattern 2 and 3), specifically up-regulated (pattern 4), and down-regulated in 25 Gy irradiation (pattern 5). **(B)** Defined clusters that reveals specific expression pattern of DEPs at day 7 (*P*-value <0.05, 1,283 proteins). The clusters were divided with 2.476 threshold. The detailed clusters show the protein alteration patterns with specifically up-regulated in 2 Gy irradiation (pattern 1), gradually increasing according to increasing dose of irradiation (pattern 2 and 3), specifically up-regulated (pattern 4), and down-regulated in 25 Gy irradiation (pattern 5).

Our study focused on proteins that were expressed differently in the 25 Gy group in comparison to 0 and 2 Gy control groups. Hence, the proteins from the 2 Gy-specific changes are not considered in this article. To further dissect proteome alterations, we classified DEPs in several patterns. As shown in Figure 3A, there were 34 proteins at day 3 showing 25 Gy-specific downregulated patterns. GO analysis showed that “intracellular signal transduction” as well as “cell to cell adhesion” were significantly (count >5 and *P*-value <0.01) affected in 25 Gy irradiated heart. One of the downregulated proteins, Mylk3, is Well-known in regulation of cardiomyocyte contractility.

In addition, 167 proteins were downregulated at day 7 in 25 Gy sample (Figure 3B). GO analysis showed that most of them belonged to the processes “Ras protein signal transduction,” “GTPase activity regulation,” or “actin filament-based process.” However, no individual protein that was downregulated at both time points (days 3 and 7) in 25 Gy sample was identified.

In contrast, 71 proteins were upregulated at day 3 in 25 Gy sample (Figure 3A). GO analysis showed that most of the upregulated proteins play an important role in “cellular stress response,” “endomembranal organization,” or “endoplasmic reticulum stress response.” At day 7, 42 proteins, associated with response to drug, organic substance, or radiation, were upregulated in 25 Gy samples (Figure 3B). It is assumed that the radiation-induced molecular biological reactions initiated immediately and proceeded during the first week after irradiation. Four proteins, consistently showed 25 Gy-specific upregulated patterns at both time points (days 3 and 7): Sulf2, Gas6, Tmem120a, and Rps27l. For example, Sulf2 can enhance infarct border-zone capillarization, and exert sustained beneficial effects on cardiac function and survival after myocardial infarction, and also known to be an essential regulators for matrix transmission and signal reception of contractile function from muscle to neurons (28, 29). Gas6 is known to be able to induce cardiac fibrosis (30). Tmem120a (Transmembrane Protein 120A) is a novel class of ion channels involved in sensing pain by transducing mechanical forces into electrical signals (31).

### Pairwise Dose Group Comparison by *t*-Test

**Figure 4:**
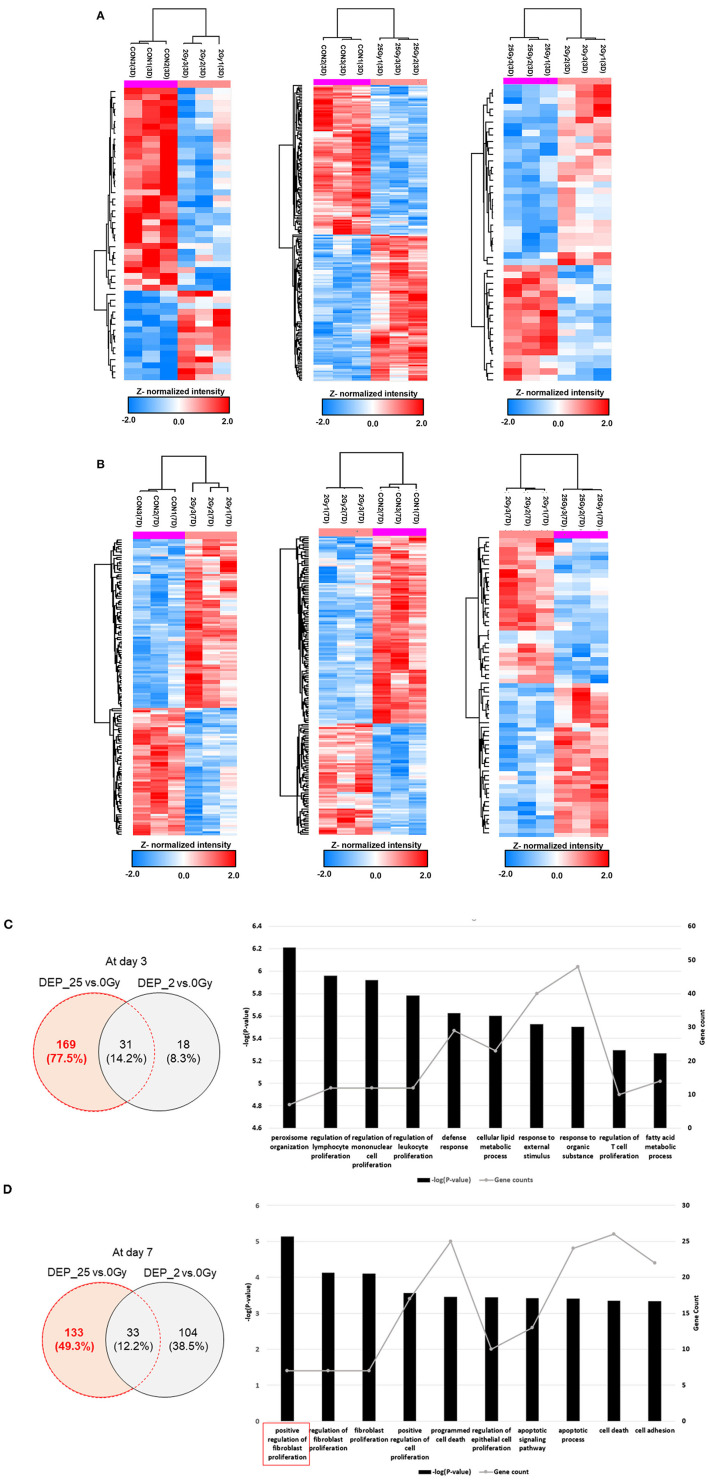
Protein expression comparison based on *t*-test between each different dose group and biological process of specifically expressed proteins at 25 Gy irradiation. **(A)** The hierarchical clustering of DEPs after *t*-test comparison between each group individually at day 3. The colorimetric scheme represented z-normalized intensity of DEPs. 49 DEPs between 0 and 2 Gy, 200 DEPs between 0 and 25 Gy, and 46 DEPs between 2 and 25 Gy. **(B)** The hierarchical clustering of DEPs after *t*-test comparison between each group individually at day 7. The colorimetric scheme represented z-normalized intensity of DEPs. 137 DEPs between 0 and 2 Gy, 166 DEPs between 0 and 25 Gy, and 68 DEPs between 2 and 25 Gy. **(C)** The 169 specifically expressed proteins at 25 Gy at day 3, determined by excluding DEPs between 0 and 25 Gy from DEPs between 0 and 25 Gy. The biological process of those were filtered with *P*-value lower than 0.05. The bar chart represent −(P - value) and spotted plot represent the number of proteins included in specific process. **(D)** The 133 specifically expressed proteins at 25 Gy at day 7, determined by excluding DEPs between 0 and 25 Gy from DEPs between 0 and 25 Gy. The biological process of those were filtered with *P*-value lower than 0.05. The bar chart represent −(P - value) and spotted plot represent the number of proteins included in specific process.

Figures 4A,B showed pairwise dose group comparison by *t*-test at each time point. The complete list of DEPs with upregulated (*P*-value <0.05 and fold-change >1.2), and downregulated (*P*-value <0.05 and fold-change <0.84) were shown in the Supplementary Table 1. Detailed analysis scheme was summarized in Supplementary Figure 4. At day 3, 200 proteins were differentially expressed after 25 Gy, of which 169 did not overlap with DEPs in control vs. 2 Gy (Figure 4C). GO-analysis of 25 Gy-specific changes displayed a process involving such as “defense response,” “response to external stimulus,” or regulations of immune cell proliferation. At day 7, 166 proteins were differentially expressed after 25 Gy, of which 133 did not overlap with changes after 2 Gy (Figure 4D). GO-analysis of these proteins demonstrated that the “positive regulation of fibroblast proliferation” was the mostly enriched biological process, which included seven proteins such as Aqp1 (Aquaporin-1) and Kng1 (Kininogen-1).

### Investigation of Specific GO:0061337 (Cardiac Conduction)

Among the 63 cardiac conduction-related proteins, six distinct proteins were differently expressed in 25 Gy group. Compared to 0 Gy group, Ryr2 (Purkinje myocyte to ventricular cardiac muscle cell signaling) was upregulated at day 3 (FC 1.315, *P*-value = 0.029) and Cav1 (regulation of heart rate by cardiac conduction) was upregulated at day 7 (FC 1.414, *P*-value = 0.004). In contrast, Cacna2d2 (Calcium Voltage-Gated Channel Auxiliary Subunit Alpha2delta2) was downregulated at day 3 (FC 0.828, *P*-value = 0.043) and Gja3 (gap junction protein) (FC 0.779, *P*-value = 0.027), Scnb2 (Sodium channel beta 2 subunit) (FC 0.796, *P*-value = 0.001), and Kcnn3 (Potassium Calcium-Activated Channel Subfamily N Member 3) (FC 0.790, *P*-value = 0.028) were downregulated at day 7.

Collectively, the aforementioned results implied that 25 Gy single fractional irradiation might be associated with the changes of cardiac conduction-related proteins by affecting various intracellular signaling pathways/ion channel proteins/actin filament-based process in terms of response to radiation stimulation, which was distinct from 2 Gy single fractional irradiation.

### Validation Phase

Among the candidate proteins showing the 25 Gy-specific changes in ANOVA, those simultaneously satisfying FC >1.2 (25 Gy compared with 0 Gy), were further included in the validation target list. In addition, several proteins in the related GO terms samples (cardiac conduction, sarcoplasmic reticulum, intercalated disk, vacuolar membrane, and cation channel activity), extracted from the previously reported histologic findings after cardiac radioablation (32), were also investigated although those proteins were not all within the 25 Gy-specific change group by ANOVA at the time of initial profiling. Therefore, the 58 candidates, except duplicated proteins, were selected (Table 1).

**Table 1:** Complete list of validation target proteins.

**#**	**Name**	**Accession**	**Description**	**Time**	**ANOVA pattern in** **profiling or candidate GO** **term**
1	Axin1	O70239	Axin-1	3 days	25-Gy specific up
2	Ccl21	Q5RJN3	C-C motif chemokine ligand 21	3 days	25-Gy specific up
3	Pdlim5	Q62920-2	Isoform 2 of PDZ and LIM domain protein 5	3 days	25-Gy specific up
4	Rab3d	Q63942	GTP-binding protein Rab-3D	3 days	25-Gy specific up
5	Gsta1	B6DYP8	Glutathione S-transferase	7 days	25-Gy specific up
6	rCG_21069	D3ZCD6	RCG21069	7 days	25-Gy specific up
7	Slc27a3	D3ZJA9	Solute carrier family 27 (fatty acid transporter), member 3 (Predicted)	7 days	25-Gy specific up
8	Myot	D3ZTC5	Myotilin	7 days	25-Gy specific up
9	Lig4	D4A0U6	DNA ligase	7 days	25-Gy specific up
10	Abcb1b	D4A0Y9	ATP-binding cassette, subfamily B (MDR/TAP), member 1B	7 days	25-Gy specific up
11	Klhdc7a	M0R439	Kelch domain-containing 7A	7 days	25-Gy specific up
12	Ephx1	P07687	Epoxide hydrolase 1	7 days	25-Gy specific up
13	Mmp2	P33436	72 kDa type IV collagenase	7 days	25-Gy specific up
14	Phlda3	Q5PQT7	Pleckstrin homology-like domain family A member 3	7 days	25-Gy specific up
15	Capg	Q6AYC4	Macrophage-capping protein	7 days	25-Gy specific up
16	Insig2	Q80UA9	Insulin-induced gene 2 protein	7 days	25-Gy specific up
17	Osbpl5	A0A0G2JV78	Oxysterol-binding protein	7 days	25-Gy specific down
18	Gtf3c3	A0A0G2K945	General transcription factor IIIC subunit 3	7 days	25-Gy specific down
19	Mid1ip1	A7BKC9	MID1 interacting G12-like protein	7 days	25-Gy specific down
20	Glb1l	B1WBS6	Galactosidase, beta 1-like	7 days	25-Gy specific down
21	Tyw5	D3ZY75	tRNA-yW synthesizing protein 5	7 days	25-Gy specific down
22	Myorg	D4AE63	Myogenesis-regulating glycosidase	7 days	25-Gy specific down
23	LOC690284	F1LTK0	Similar to F49E2.5d	7 days	25-Gy specific down
24	Kcnn3	G3V8S7	Potassium intermediate/small conductance calcium-activated channel, subfamily N, member 3, isoform CRA_a	7 days	25-Gy specific down
25	Ftl1	P02793	Ferritin light chain 1	7 days	25-Gy specific down
26	Agap2	Q8CGU4	Arf-GAP with GTPase, ANK repeat and PH domain-containing protein 2	7 days	25-Gy specific down
27	Sulf2	Q3L472	Extracellular sulfatase	3 & 7 days	ANOVA 25-Gy specific up
28	Gas6	Q63772	Growth arrest-specific protein 6	3 & 7 days	ANOVA 25-Gy specific up
29	Tmem120a	Q5HZE2	Transmembrane protein 120A	3 & 7 days	ANOVA 25-Gy specific up
30	Rps27l	P24051	40S ribosomal protein S27-like	3 & 7 days	ANOVA 25-Gy specific up
31	Wnt5a	Q9QXQ7	Protein Wnt-5a	7 days	*T*-test top GO term (positive regulation of fibroblast proliferation)
32	Kng1	A0A0G2KA54	Kininogen-1	7 days	*T*-test top GO term (positive regulation of fibroblast proliferation)
33	Kng1	P08934	Kininogen-1	7 days	*T*-test top GO term (positive regulation of fibroblast proliferation)
34	Cdkn1a	Q64315	Cyclin-dependent kinase inhibitor 1A	7 days	*T*-test top GO term (positive regulation of fibroblast proliferation)
35	Aqp1	P29975	Aquaporin-1	7 days	*T*-test top GO term (positive regulation of fibroblast proliferation)
36	Cd74	P10247	H-2 class II histocompatibility antigen gamma chain	7 days	*T*-test top GO term (positive regulation of fibroblast proliferation)
37	Ryr2	B0LPN4-2	Isoform 2 of ryanodine receptor 2	NA	Specific GO (cardiac conduction)
38	Cav1	A2VCW2	Caveolin (fragment)	NA	Specific GO (cardiac conduction)
39	Cacna2d2	A0A0G2K0J2	Voltage-dependent calcium channel subunit alpha-2/delta-2	NA	Specific GO (cardiac conduction)
40	Gja3	G3V747	Gap junction protein	NA	Specific GO (cardiac conduction)
41	Scnb2	Q62861	Sodium channel beta 2 subunit	NA	Specific GO (cardiac conduction)
42	Atp2a1	Q64578	Sarcoplasmic/endoplasmic reticulum calcium ATPase 1	NA	Specific GO (sarcoplasmic reticulum)
43	Camk2g	P11730	Calcium/calmodulin-dependent protein kinase type II subunit gamma	NA	Specific GO (sarcoplasmic reticulum)
44	Actn1	Q9Z1P2	Alpha-actinin-1	NA	Specific GO (intercalated disk)
45	Anxa5	P14668	Annexin A5	NA	Specific GO (intercalated disk)
46	Scn4b	Q7M730	Sodium channel subunit beta-4	NA	Specific GO (intercalated disk)
47	Abca2	G3V7X4	ATP-binding cassette sub-family A member 2	NA	Specific GO (vacuolar membrane)
48	Abcd1	D3ZHR2	ATP-binding cassette subfamily D member 1	NA	Specific GO (vacuolar membrane)
49	Cd68	Q4FZY1	Cd68 molecule	NA	Specific GO (vacuolar membrane)
50	Slc39a14	D3ZZM0	Solute carrier family 39 (Zinc transporter), member 14 (Predicted)	NA	Specific GO (vacuolar membrane)
51	Atp5b	Q0QEP3	ATP synthase, H+ transporting mitochondrial F1 complex, beta subunit	NA	Specific GO (cation channel activity)
52	Calhm5	Q5FWS4	Calcium homeostasis modulator family member 5	NA	Specific GO (cation channel activity)
53	Cx43		Connexin-43	NA	Specific GO (cardiac conduction)
54	Scn5a		Sodium channel subunit 5a	NA	Specific GO (cardiac conduction)
55	cTnT		Cardiac troponin T	NA	Specific GO (cardiac conduction)
56	Kcnh2		hERG K channel	NA	Specific GO (cardiac conduction)
57	Kcnd3		Transient outward K channel	NA	Specific GO (cardiac conduction)
58	Cacna1c		L-type Ca channel	NA	Specific GO (cardiac conduction)

**Figure 5:**
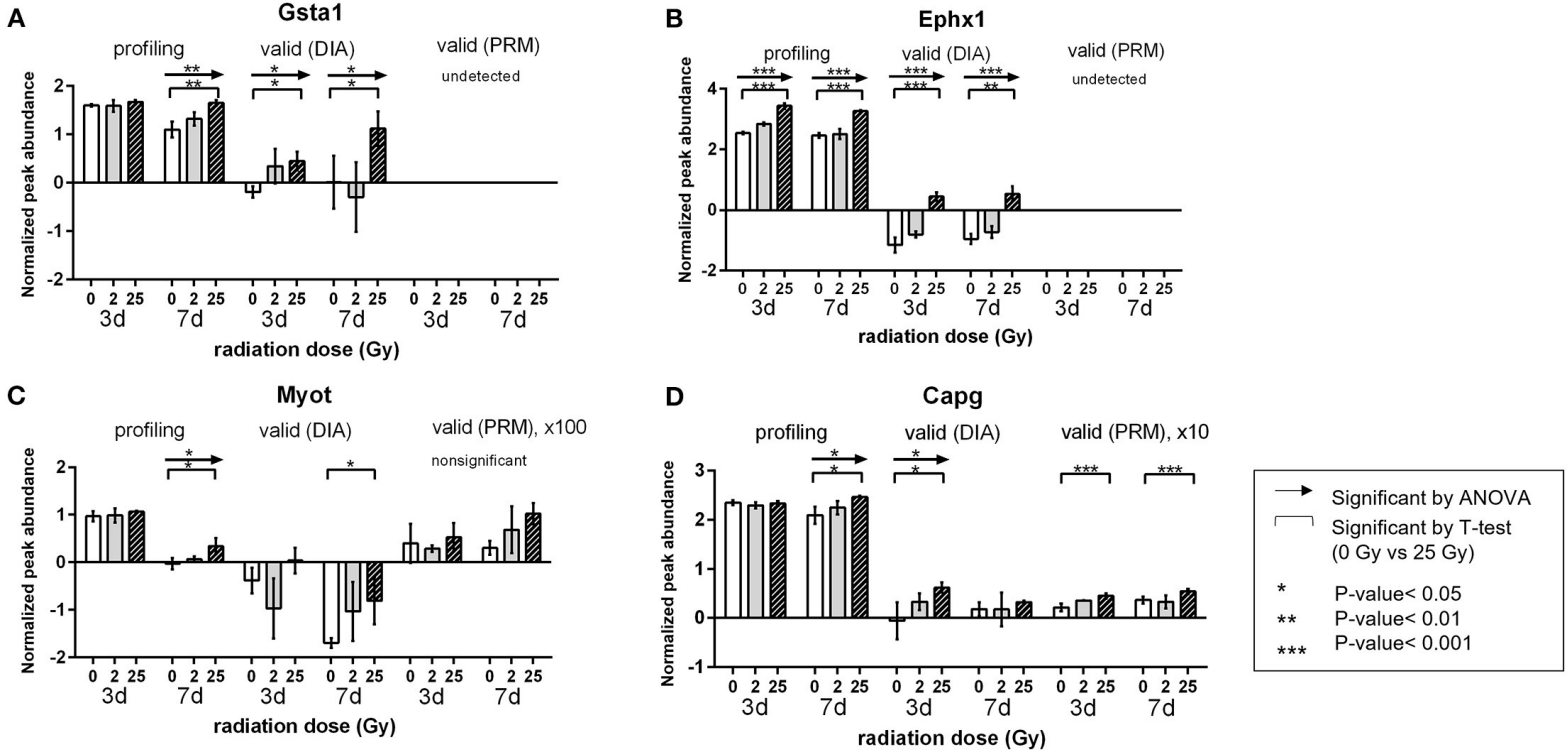
Validation results of the candidate proteins. Changes in expression of each protein by radiation dose and timepoint. Comparison was done within each validation method. **(A)** Gsta1 **(B)** Ephx1 **(C)** Myot **(D)** Capg. Relative amount is a value of log_2_ normalized quantified in profiling and DIA method. *P < 0.05, **P < 0.01, ***P < 0.001.

Detailed validation results of the candidate proteins were included in Supplementary Table 2. After validation, the PRM analysis, which is the targeted quantification method detected 19 proteins and demonstrated that 13 proteins among those presented statistically significant changes (ANOVA-test, *P*-value <0.05). In similar, DIA detected 21 proteins and verified 10 proteins expressed significant protein alterations (ANOVA-test, *P*-value <0.05). According to the validation result, we investigated the final candidates that were statistically verified from at least one of the validation process and the protein expressions were significant on 25 Gy. As a result, four proteins (Gsta1, Myot, Ephx1, and Capg) were repeatedly detected showing 25 Gy-specific up patterns at day 7 (Figure 5). Specifically, Gsta1 (Glutathione S-Transferase Alpha 1) (33, 34) and Ephx1 (Epoxide hydrolase 1) (35, 36) are detoxifying enzymes involved in the recovery process after cardiac injury. Myot is a sarcomeric protein that binds and cross-links actin filaments, and contributes to filaments stabilization (37). Capg is an actin regulatory protein from cardiac macrophages with critical role in maintaining cardiac conduction (38, 39). Interestingly, their role in arrhythmia modulation have not been investigated.

All initially profiled proteins showing 25 Gy-specific down regulated pattern were not identified in validation experiments. Other selected validation targets linked to ion channel or cell junction (Cx43: FC 0.818, Scn5a: FC 0.846, and cTnT: FC 0.442) showed decreased expression in the 25 Gy group compared to that with 0 Gy at day 3, but those trends were not clearly observed at day 7. However, these results could not be detected during the initial profiling (Supplementary Figure 5). Moreover, we also investigated the validation result of 63 DEPs related with cardiac conduction (specific GO:0061337) as like in profiling phase (Supplementary Table 3). Although only 11 proteins were identified, Ryr2 and Cav1 that were already mentioned in the above result section were included, which showed the early dynamics of these proteins. Therefore, our validation results have to be interpreted with caution and further robust experimentation evaluating these questions need to be performed.

Based on the aforementioned results, Gsta1, Myot, Ephx1, and Capg may be one of the potential clues that can explain for SABR-induced early antiarrhythmic effects. As these proteins are involved in stress-induced innate responses or cardiac remodeling processes, further exploration about the role on anti-arrhythmic effects is needed.

## Discussion

The present study characterized the proteomic changes after single dose irradiation to the normal rat heart. To the best of our knowledge, this is the first such proteomic study to describe biological changes caused by single high-dose irradiation (25 Gy). The DEPs after statistical analysis were mostly related with cardiovascular system development such as actin filament-based process, supramolecular fiber organization, or muscle contraction. The protein expressions among those biological process was down regulated from both of day 3 and 7 after 25 Gy, or some of those were upregulated on day 3, but eventually half of those proteins become downregulated after day 7. According to reported study that actin filament or fiber organization are related with wound healing or tissue regeneration (40), our proteomic results are expected to reflect a favorable prognosis. In addition, the KEGG pathway analysis result also suggested equivalent aspects. Most of proteins on regulation of actin cytoskeleton and VEGF signaling pathway are also downregulated after 25 Gy irradiation on both of day 3 and 7. Although, Camk2g (calcium/calmodulin-dependent protein kinase type II subunit gamma) on TB pathway, that regulates various downstream signal which promotes heart failure, arrhythmias, or vascular related disease (41), was upregulated at day 3 after 25 Gy, its expression decrease at day 7.

Since we focused only on the proteins showing 25 Gy-specific changes differentially expressed from the two control groups with sufficient FC (>1.2), the number of targets requiring validation were reduced and selected the candidates that are validated by at least one LC-MS/MS analysis among DIA and PRM method. Finally, only four proteins that showed 25 Gy-specific increase at day 7 were confirmed by independent experimentation. Two proteins, Gsta1 and Ephx1, are associated with defense responses suggesting a radiation-induced activation of innate defense mechanism might also neutralize abnormal conduction status as an unintended effect. Gsta1 also possesses cardioprotective property through scavenging free radicals and increasing resistance to ROS (42). The other two proteins, Myot and Capg, are associated with cytoskeleton stabilization and remodeling but the association between cytoskeletal protein function and cardiac electrical activity remains unclear in cardiac arrhythmia (43). A few recent studies reported potential links between cytoskeletal microenvironment and arrhythmia that may contribute to antiarrhythmic effects (38, 39). Hence, as our suggested candidates of SABR-induced mediator of early cardiac effects are not known to be directly related to the common mechanism of arrhythmias, further research may be required to understand their specific antiarrhythmic mechanism of action.

We performed DEP analysis on 63 cardiac conduction-related proteins assuming that the early effect of cardiac radioablation would change cardiac conduction proteins expression. DEP analysis showed that Ryr2 and Cav1 were upregulated and Cacna2d2, Gja3, Scnb2, and Kcnn3 were downregulated in the 25 Gy group compared to the controls. The validation process among those DEPs resulted in that 19 proteins were detected and 11 proteins were statistically significant from overall of DIA and PRM analysis (Supplementary Table 3). The Ryr2 and Cav1 that were upregulated from profiling analysis were validated from ANOVA test by DIA method in day 3 and *t*-test that compared 0 and 25 Gy from day 7 by PRM method, respectively. However, Cacna2d2, Gja3, Scnb2, and Kcnn3 that were downregulated were not detected or statistically significant. Moreover, most of the results were not replicated in the validation phase. By DIA or PRM methods, expression of Cx43, Scn5a, and cTnT was decreased in the 25 Gy group at day 3 but resolved by day 7. Likewise, several proteins drew attention as the cause of radioablating effects, but no study evaluating these in the early 7 days after high-dose irradiation exists. Connexin-43 expression by immunofluorescent staining decreased at 2–3 weeks, but slightly increased at 4 weeks after 30 Gy irradiation in rat heart in our previous study (32). Viczenczova et al. (44) demonstrated that 25 Gy single irradiation increased total Cx43 in rat myocardium after 6 weeks. Zhang et al. (7) also identified RT-induced increase of Na_v_1.5 (voltage-gated sodium channel) and Cx43 in murine heart after 6 weeks of irradiation. Note that it is difficult to draw a consistent conclusion because the evaluation time point and observational methods are different for each study, but it could be an area that needs attention.

Different mechanisms have been postulated as the basis of an antiarrhythmic effect of SABR, such as the destruction of the focus, modulation of the conduction activity, or traditionally expected fibrosis (7, 45, 46). The effects of cardiac radioablation are assumed to be due to radiation-induced fibrosis which could block conduction within an aberrant circuit. However, clinical observations showing the rapid reduction of VT episode within few days after radioablation suggested that the underlying mechanism is dependent on more than fibrosis. Cha et al. (32) described early pathological changes after cardiac radioablation in rat, suggesting interstitial edema and intercalated disc widening may play a more important role rather than necrosis or fibrosis in early phase. Meanwhile, one recent human study evaluated the explanted recipient heart from four patients after cardiac radioablation and reported that subendocardial necrosis surrounded by a rim of fibrosis, myointimal thickening, or irregular, convoluted intercalated disc regions were indicative of cellular injury after SABR and not observed in other areas that were not treated (47). The authors postulated that while short-term effects were induced by damage to the rapid turnover machinery, long-term effects were caused by fibrosis. In our pairwise group comparison by *t*-test, 25 Gy sample displayed processes such as “defense response” and “response to external stimulus” at day 3, and “positive regulation of fibroblast proliferation” at day 7. While further investigation is warranted, the results of our study provide a preliminary insight into various mechanisms that change their contribution in disease progression over time.

This study has several limitations. First, our experimental rat model was not an arrhythmia-specific model, so the observed radiation response of the normal heart might be different from the pathological response. Additionally, radiation response of human tissue could be different from that of rat tissue. Second, down-regulated targets after cardiac radioablation are difficult to detect and may not be verified by proteomics. Intrinsic complexity and non-specificity of radiation response might affect the validation results dependent on the identification methods. Interestingly, several proteins of interest, associated to ion channel or cell junction pathways, showed decreased expression after 25 Gy; however, these results were not confirmed in either experimentation. Compared to abundant cytoskeletal proteins, detection of ion channel proteins may have been limited due to low cellular concentrations. Unfortunately, additional electrophysiologic data and experimentation had not been performed concomitantly which would be required to satisfactorily conclude. It is also regrettable that we were not able to additionally conduct a mechanism study on a specific candidate protein we found. Future technological advances and the application of integrative approaches may provide a more comprehensive understanding of high-dose radiation-related alterations in cardiac conduction tissues.

In conclusion, single high-dose irradiation induces large proteomic changes within a week of exposure and several targets were identified as potential candidates of mediating early antiarrhythmic effects of radioablation. It seems to be helpful in establishing a hypothesis for the rapid effects observed in clinical studies, and a comprehensive analysis effort is needed to determine whether a relation to histologic changes exists. Further detailed investigation into a role of suggested candidates in the conduction tissue will be needed.

## Data Availability Statement

The datasets presented in this study can be found in online repositories. The names of the repository/repositories and accession number(s) can be found below: https://www.ebi.ac.uk/pride/archive/, PXD030878

## Ethics Statement

The animal study was reviewed and approved by the Institutional Animal Care and Use Committee at the Seoul National University Hospital (IACUC No. 18-0245-S1A0).

## Author Contributions

BK, M-JC, and JC conceived the experiments. BK, JJ, DH, M-JC, and JC performed the experiments and analyzed the data. JJ and DH performed bioinformatical analysis. M-JC and JC supervised the project. BK, DH, M-JC, and JC wrote the manuscript. All authors assisted with manuscript preparation, discussed the manuscript, commented on the project, and contributed to manuscript preparation.

## Funding

This work was supported by a general clinical research grant-in-aid from the Seoul Metropolitan Government Seoul National University (SMG-SNU) Boramae Medical Center (03-2020-21) to BK, a grant from the National Research Foundation (NRF) of Korea funded by the Korean government (No. NRF-2020R1A2C1013832) to M-JC and (No. NRF-2018M2A2B3A01070410) to JC.

## Conflict of Interest

The authors declare that the research was conducted in the absence of any commercial or financial relationships that could be construed as a potential conflict of interest.

## Publisher's Note

All claims expressed in this article are solely those of the authors and do not necessarily represent those of their affiliated organizations, or those of the publisher, the editors and the reviewers. Any product that may be evaluated in this article, or claim that may be made by its manufacturer, is not guaranteed or endorsed by the publisher.

## Supplementary Material

Supplementary Figure 1 Overall experimental scheme of quantitative proteomic analysis.

Supplementary Figure 2 Label-free analysis strategy of the harvested tissues.

Supplementary Figure 3 **(A)** Coefficient variance (CV) distribution after width adjustment normalization of protein abundance from each sample. **(B)** Dynamic range of protein abundance of rat heart proteome. The iBAQ intensities of each sample were used with top and bottom of five abundance proteins were marked. **(C)** Principle-component analysis (PCA) plot comparison between radiation doses.

Supplementary Figure 4 Detailed dose group comparison scheme after LC-MS/MS analysis. The differentially expressed proteins (DEPs) after 0, 2, and 25 Gy irradiation were identified after pairwise student *t*-test. The DEPs were calculated with fold change lower than 0.84 or greater than 1.2 with *P*-value lower than 0.05.

Supplementary Figure 5 Validation results of candidate proteins. **(A)** Cx43 **(B)** cTnT **(C)** Scn5a. Changes in expression of each protein by radiation dose and timepoint. Comparison was done with each validation method.
