# Supplementary material for: One-Week Dynamic Changes in Cardiac Proteomes After Cardiac Radioablation in Experimental Rat Model
Source: Front Cardiovasc Med. 2022 Jun 28;9:898222. doi: 10.3389/fcvm.2022.898222 (PMC9273889; doi:10.3389/fcvm.2022.898222)

Supplemental Figure 1

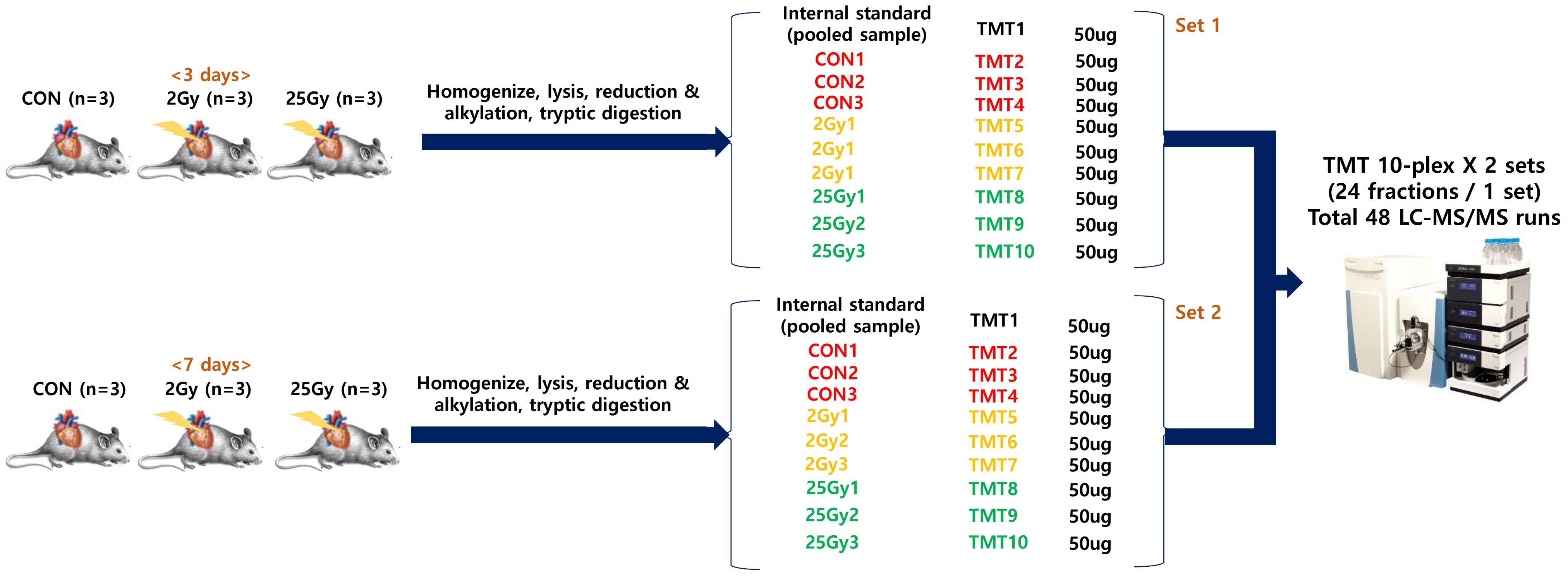

Supplemental Figure 2

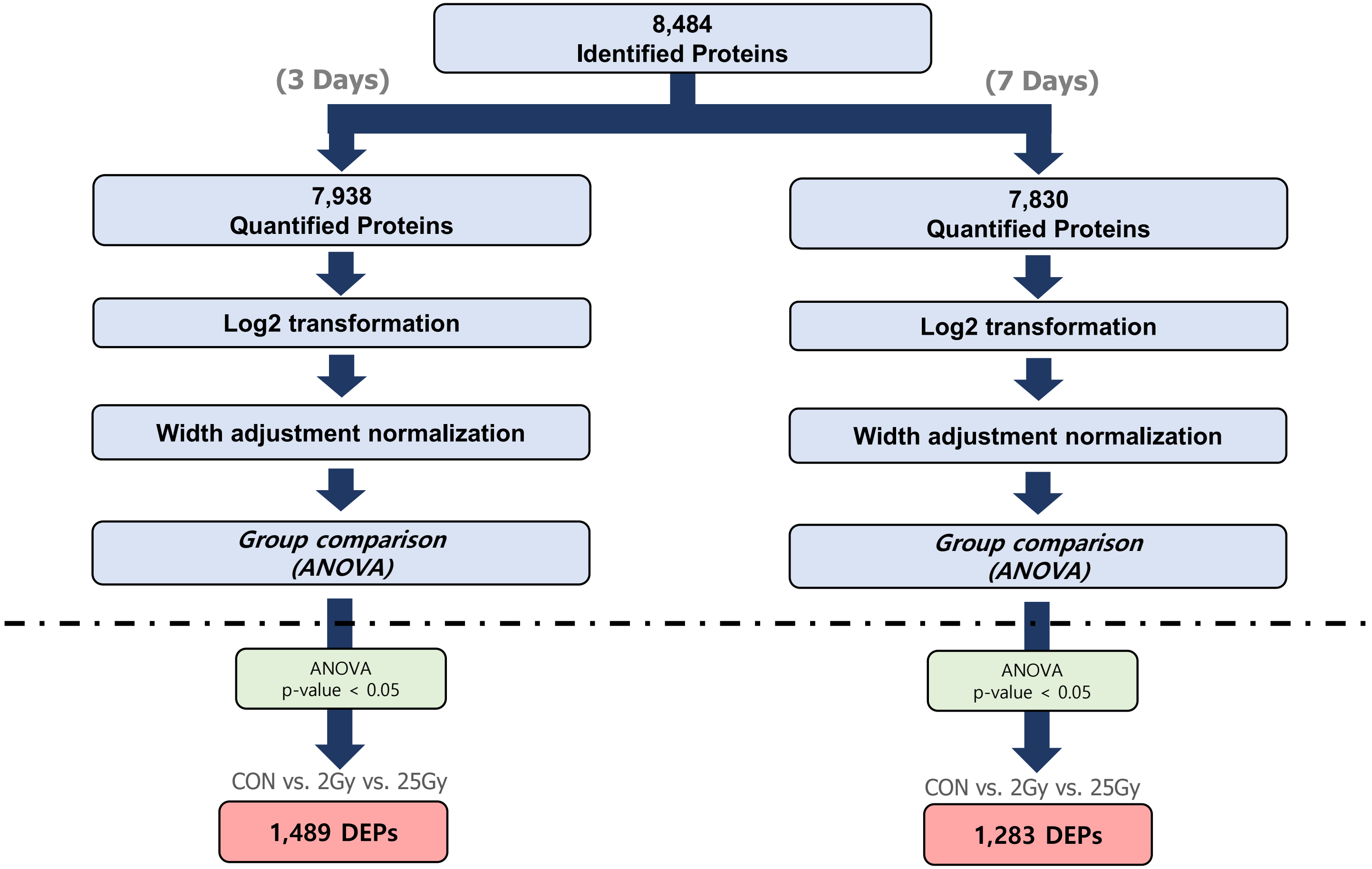

Supplemental Figure 3

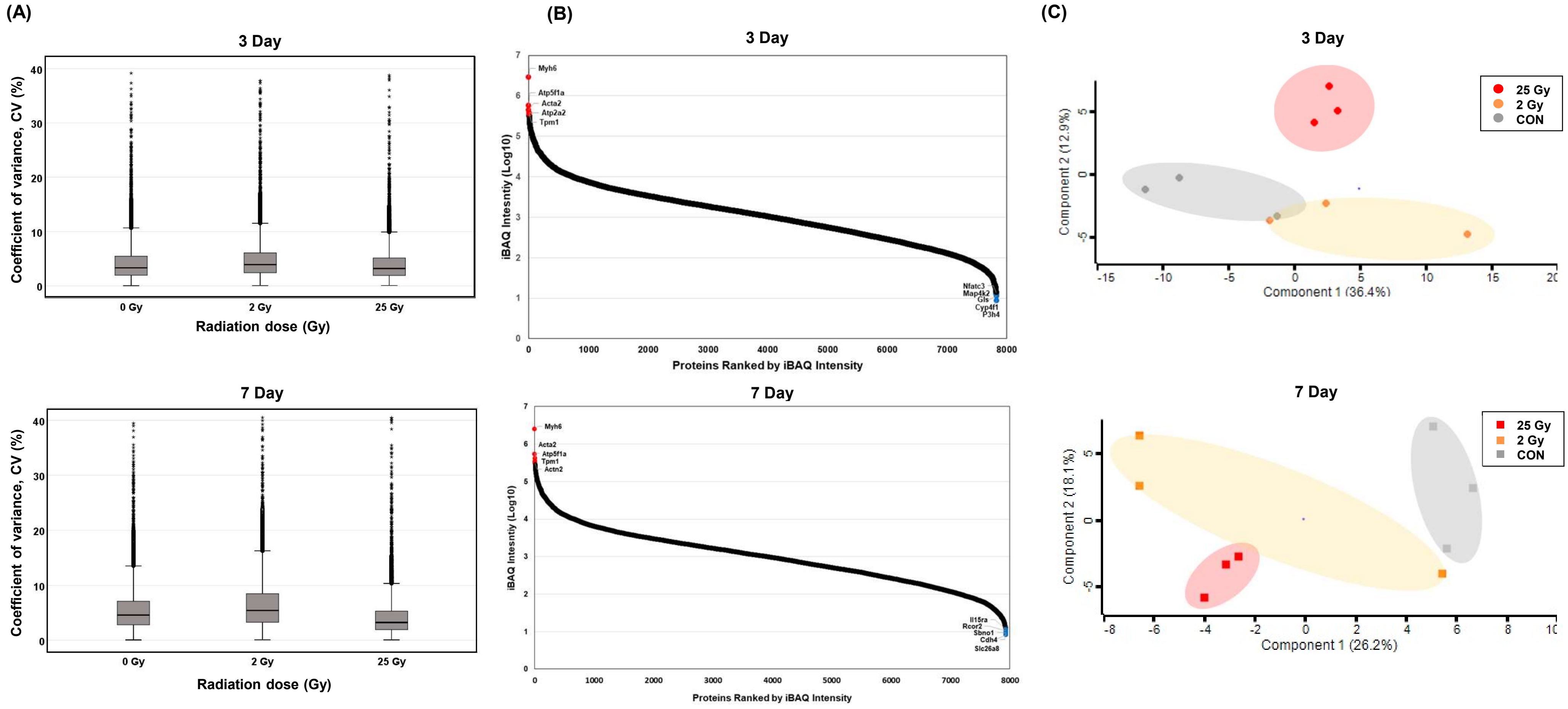

Supplemental Figure 4

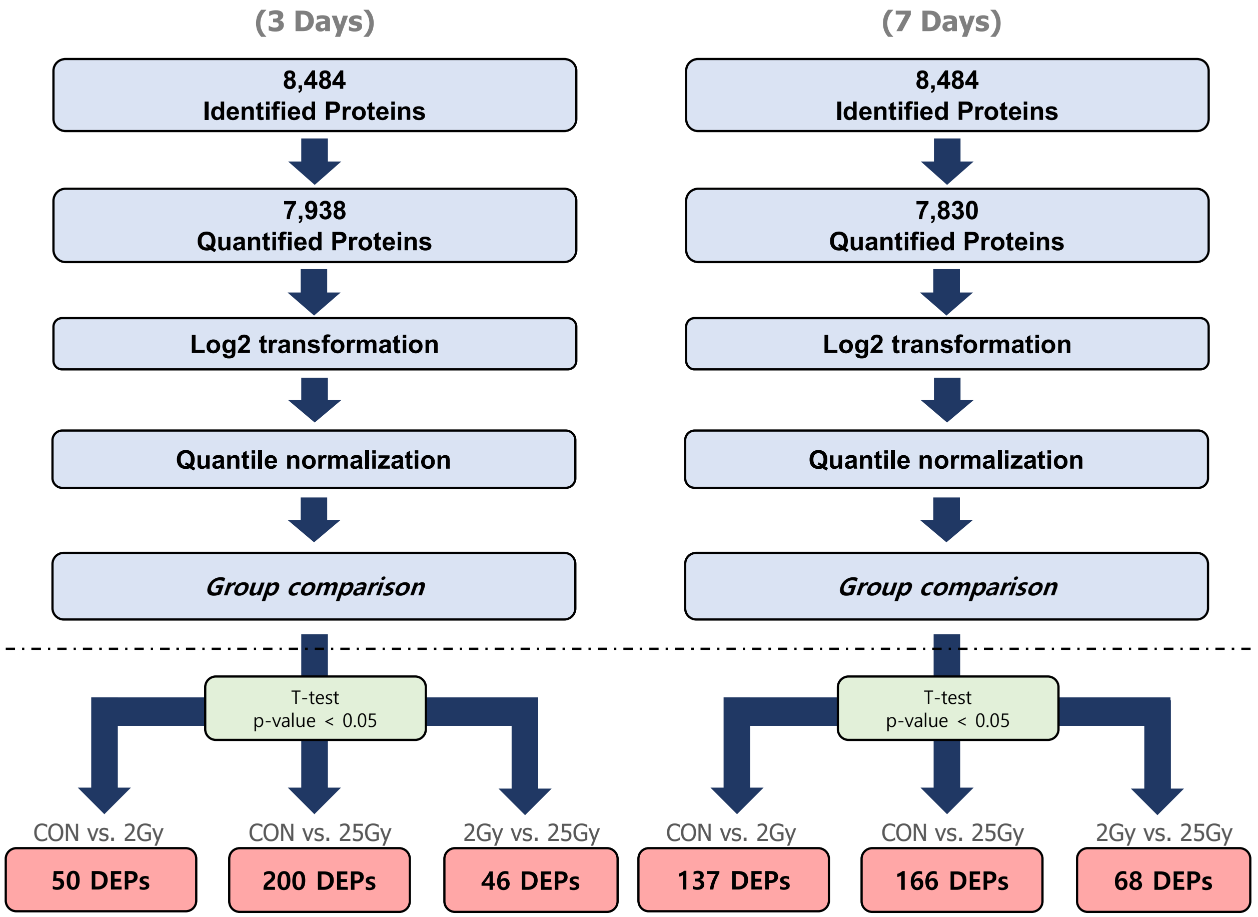

Supplemental Figure 5

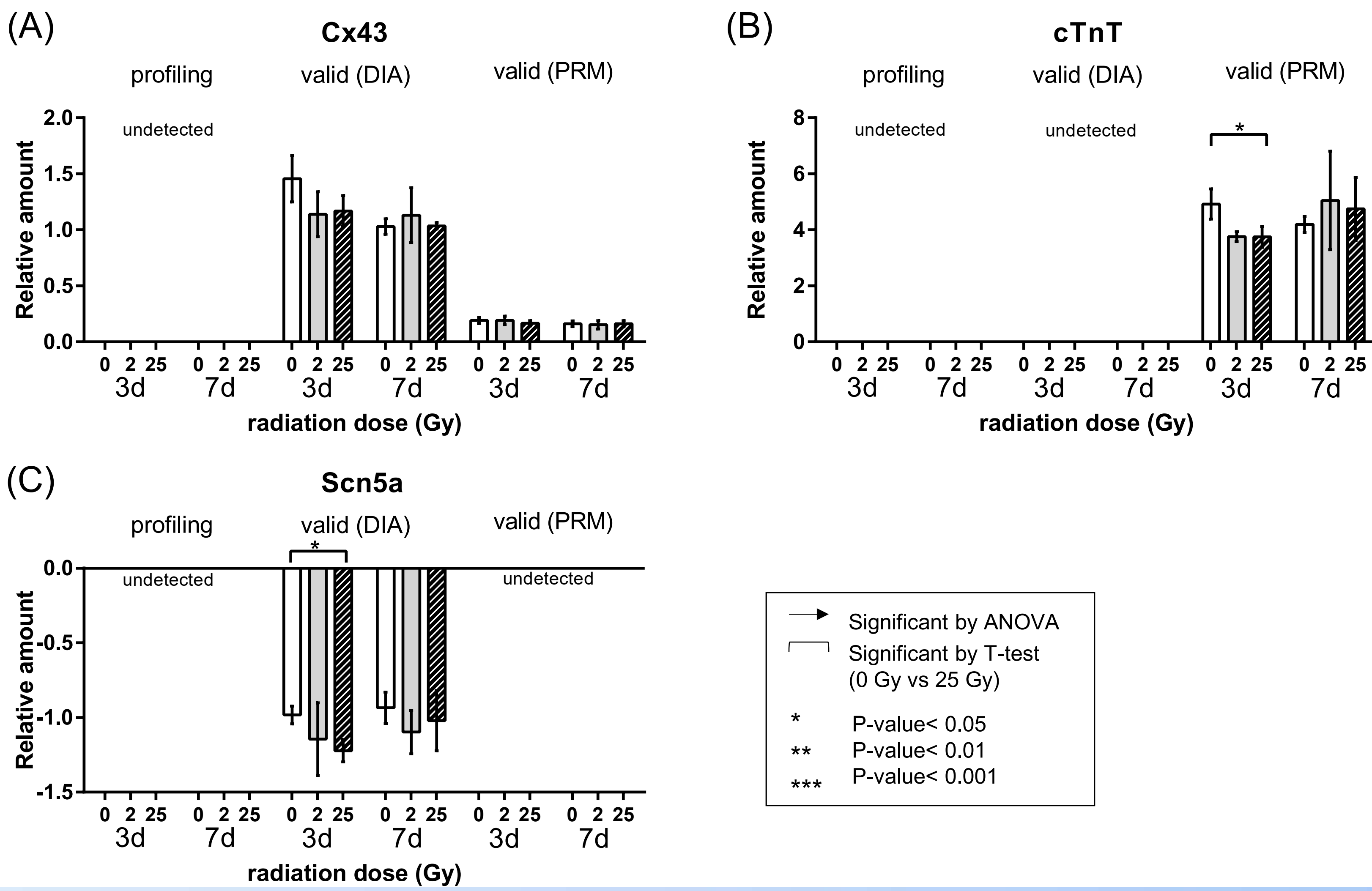

Supplement: Supplementary Figure 1 — Overall experimental scheme of quantitative proteomic analysis. [file Data_Sheet_2.PDF]
