# Supplementary material for: One-Week Dynamic Changes in Cardiac Proteomes After Cardiac Radioablation in Experimental Rat Model
Source: Front Cardiovasc Med. 2022 Jun 28;9:898222. doi: 10.3389/fcvm.2022.898222 (PMC9273889; doi:10.3389/fcvm.2022.898222)
Supplement: Supplementary file 5 [file Data_Sheet_1.DOCX]

**Sample preparation**

Heart tissue samples were prepared using a filter-aided sample preparation (FASP) method modified for frozen tissue preparation (PMID:33453410). First, frozen tissue samples were homogenized with lysis buffer (4% SDS, 2 mM tris(2-carboxyethyl)phosphine, and 0.1 M Tris-HCl, pH 7.4). The protein concentration was determined by tryptophan fluorescence emission at 350 nm using an excitation wavelength of 295 nm (PMID: 25837572). Proteins were digested using the 2-step FASP procedure with some modifications (PMID: 29950347; PMID:33453410). To remove contaminants, we performed acetone precipitation at −20 °C with 200 μg of the lysate. Pellets were resuspended in SDT buffer (2% SDS, 10mM TCEP, and 50mM CAA in 0.1M Tris pH 8.0). Then, the solution loaded onto a 30K Amicon filter (Millipore, Burlington, MA, USA). The buffer was exchanged to UA solution (8M urea in 0.1M Tris pH 8.5) using centrifugation at 14,000 g. Following the exchange of 50 mM HEPES buffer, proteins were digested at 37°C overnight using a trypsin/LysC mixture (protein-to-protease ratio of 100:1). The peptides were isolated using centrifugation. After the filter units were rinsed with 50 mM HEPES, we performed second digestion at 37°C for 2 hours using trypsin (enzyme-to-substrate ratio [w/w] of 1:1,000). The digested peptides were acidified using 10% trifluoroacetic acid and desalted using homemade C18-StageTips as previously described (PMID: 29950347; PMID:33453410). Finally, we used a vacuum dryer to dry it and stored at −80°C.

**TMT 10-plex labelling**

Tandem mass tag (TMT) labelling was performed according to the manufacturer’s protocol with some modifications (PMID: 34707096). Briefly, TMT 10-plex (Thermo Fisher Scientific, Waltham, MA, USA) reagent (0.8 mg) was dissolved in 100% ACN. Each 20-µg sample was spiked with 260 ng of peptides derived from ovalbumin for use as an internal standard, and ACN was added to the reagent to give a final concentration of 30% (v/v). After incubation at room temperature for 1 h, the reaction was quenched with 5% hydroxylamine. The TMT-labeled peptides were pooled at a 1:1:1:1:1:1:1:1:1:1 ratio, and the mixtures were dried in a speed vacuum.

**Offline High-pH peptide fractionation**

For spectral library construction and TMT 10-plex quantification, peptide samples were fractionated offline using the reversed-phase high-pH strategy as described previously (PMID: 29338754; PMID:34707096). Before high-pH fractionation, the pooled peptides were desalted using Oasis solid phase extraction (SPE) columns (Waters, Milford, MA, USA), and the resulting peptides were subjected to Agilent 1290 bioinert HPLC (Agilent, Santa Clara, CA, USA) equipped with an analytical column (4.6 × 250 mm, 5 μm). For peptide separation, buffer A consisted of 15 mM ammonium hydroxide, and buffer B consisted of 15 mM ammonium hydroxide in 90% ACN. The peptides were fractionated with a gradient from 5 to 35% ACN at a flow rate of 0.2 ml/min. A total of 96 fractions were concatenated into 24 fractions and evaporated in a speed vacuum.

**LC-MS/MS analysis**

All samples for TMT 10-plex and Data independent acquisition (DIA) quantification were analyzed by LC–MS/MS using quadrupole orbitrap mass spectrometers, Q-Exactive HF-X (Thermo Fisher Scientific, Waltham, MA, USA) coupled with an Ultimate 3000 RSLC system (Dionex, Sunnyvale, CA, USA) consisting of EASY-Spray™ LC columns with an electrospray source, and the temperature of the column heater was set to 60 °C (PMID: 29338754;PMID:33453410). Peptides were separated on a two-column system with a trap column (5 mm in length and 300 µm in diameter) and an analytic column (EASY-Spray C18, 75 µm I.D. × 50 cm length, 2 µm). A gradient was applied using 0.1% formic acid in water as solvent A and 0.1% formic acid in ACN as solvent B. After the samples were loaded onto the nano LC, the samples were run with a 90 minutes gradient from 8% to 30% solvent B.

For spectral library construction, the mass spectrometer was operated in data-dependent acquisition mode. The survey MS scan (350 to 1650 m/z) was acquired at a mass resolution of 70,000 at m/z 200, and the MS/MS spectrum was acquired at a mass resolution of 17,500 at m/z 200. A tandem mass spectra of the 15 most abundant peaks were acquired by peptide fragmentation using high collision dissociation (HCD). The normalized collision energy (NCE) was set to 28% with an isolation window of 1.2 m/z.

In the cases in which quantification was based on TMT, the 20 most abundant peptide ions in the full MS scan were also fragmented using a higher HCD (NCE 32% with isolation width of 1.2 m/z).

For DIA quantification, the spray voltage was set to 2.0 kV in the positive ion mode, and the temperature of the heated capillary was 320°C. The MS method consisted of a survey scan at 35,000 resolution from 400 to 1,220 m/z. Automatic gain control (AGC) target of 3 × 10^6^ at injection time of 60 ms. Then, 19 DIA windows were acquired at 35,000 resolution with AGC target 3 × 10^6^ and auto for injection time (PMID: 25724911). Stepped collision energy was 10% at 27%.

**Data processing for spectral library construction**

For DIA and PRM, we processed MS raw files that obtained from 24 data-dependent acquisition (DDA) measurements of the pooled samples using MaxQuant (version 1.6.1.0). MS/MS spectra were searched against the Rat Uniprot reference database (September 2018, 37,316 entries) and the Biognosys iRT peptides fasta database using the Andromeda. Data was searched with 6 ppm precursor ion tolerance for total protein level analysis and 20 ppm MS/MS ion tolerance. We used variable modifications (N-acetylation of protein and oxidation of methionine) and a fixed modification (cysteine carbamido-methylation). Enzyme specificity was set as full tryptic digestion. Peptides with a minimum length of 6 amino-acids and up to 2 missed-cleavages were considered. The required false discovery rate (FDR) was set to 1% at the peptide, protein, and modification level. To maximize the number of quantification events across samples, we enabled the ‘Match between Runs’ function on the MaxQuant platform. MaxQuant search results were imported as spectral libraries into Spectronaut Pulsar version 14 with default settings.

**Data processing** **for TMT data**

MS raw files were processed using Proteome Discoverer version 2.4 with the SEQUEST-HT algorithm against the Rat Uniprot reference database (September 2018, 37,316 entries). The search parameters included full enzyme digestion using trypsin with up to two missed cleavages, 20 ppm of peptide precursor mass tolerance, and 0.02 Da of fragment ion mass tolerance. Variable modifications of 15.995 Da for methionine oxidation and 42.011 Da for protein N-terminal acetylation and fixed modifications of 57.021 Da for carbamidomethylation on cysteine residues and 229.153 Da for TMT-labeled lysine and any N-terminus were selected. The co-isolation threshold for quantification of peptides was set to 50%. The FDRs of peptide-spectral matches and proteins were set to <1%.

**Data processing for DIA data**

The DIA data of individual samples were analyzed with Spectronaut Pulsar version 14 (Biognosys). We converted the DIA raw files into an htrm format by using the GTRMS converter provided by the Spectronaut software. The FDR was estimated with the mProphet (PMID:21423193) approach and set to 1% at peptide precursor level and at 1% at protein level. The proteins were inferred by the software, and the quantification information was acquired at the protein level by using the q-value < 0.01 criteria, which was used for subsequent analyses

**Parallel Reaction Monitoring (PRM) analysis**

After proteome profiling analysis with tandem MS spectrometry, we validated the results parallel reaction monitoring (PRM). Before analysis, we prepared the stable isotope standards (SIS) peptides which contains exact equivalent amino acid sequences with unique peptides of target proteins. Those were synthesized with heavy labelled isotope of carbon and nitrogen (13C and 15N) on arginine or lysine (JPT peptide Technologies GmbH, Berlin, Germany). Those sequences were selected based on SRMAtlas database (http://www.srmatlas.org) which archived the transition data analysed with quadrupole-orbitrap MS and from in-house rat heart spectral library. We selected at least three unique peptides per each target protein. The digested peptides from rat heart tissue samples were dissolved in 0.1% formic acid and 5 ug of samples along with 200 fmol of SIS peptides were injected. In case of PRM-MS, orbitrap exploris 480 (Thermo Fisher Scientific, Waltham, MA, USA) coupled with an Ultimate 3000 RSLC system (Dionex, Sunnyvale, CA, USA) consisting of EASY-Spray™ LC columns were applied which are same setting from profiling analysis. During analysis, the method duration and linear gradient were also equivalent with those from profiling analysis. However, the method was converted as PRM tab coupled with full scan within xcalibur software. PRM method was performed with following parameters: the full scan was acquired by 70,000 resolutions with automatic gain control (AGC) target value of 3e6 and PRM properties were 17,500 resolutions with AGC target value of 1e6. Isolation window was set as 2 m/z and normalized collision energy (NCE) was differentially optimized through Skyline software depends on peptide sequences. The chromatogram peak width was set to 30 s and the other parameters were same as profiling analysis. The acquired MS data such as peak area integration, ratios, coefficient variance (CV), and retention times were manually adjusted within Skyline software [1]. The statistical analysis that compare ratios between time interval and conditions were performed with MSstats (v3.13.7) [2] which embedded in Skyline.

**Statistics analysis**

Perseus software was used for all statistical analyses of TMT data (PMID: 27348712). Reporter ion intensities were log2-transformed. After the data were normalized using width adjustment in Perseus software, ANOVA or two-sample t-tests were performed using permutation-based FDR and a significance level of 5%. For clustering analysis, normalized protein abundance levels were subjected to further z-normalization followed by hierarchical clustering in terms of the Euclidean distance and average linkage. After profiling analysis, we verified selected DEPs by DIA analysis. The statistical analysis was also performed with Perseus software. Protein intensities obtained from DIA analysis were log2 transformed for further manipulation and filtered proteins which are detected less than 70% of the total samples. Reverse and contaminant detections were subsequently removed. Missing values were replaced using the normal distribution imputation method with default settings to develop reliable differentially expressed proteins (DEPs). Those filtered proteins were normalized by width adjustment which subtract the second quartile value (q2) from each value and center the distribution by asymmetric way. After normalization, we performed multiple sample test; ANOVA test and student t-test for protein expression changes. The proteins with significant expression changes were determined with p-value lower than 0.05 and fold change lower than -1.2 or higher than +1.2 ($\mathrm{Log}_{2}FC\geq-1.2 or Log_{2}FC\leq1.2$).

**Bioinformatics analysis**

To investigate the functional features of DEPs from ANOVA and student t-test, gene ontology (GO) analysis was implemented to determine molecular function or biological process by using DAVID tool (version 6.8) [3]. Among the results from functional analysis, we categorized those with specific GO terms based on protein expression patterns and filtered by p-value (<0.05), which are related with cardiac activity and ion channel transfer activity. The PPI (protein-protein interaction) network analysis was performed with Cytoscape software (version 3.7.2) [4] and interaction database was accessed by STRING (Search Tool for the Retrieval of Interacting Genes) (version 10) was applied [5]. The expression alternation of DEPs were also displayed as colorimetric scheme of Log2 fold change values; blue for down-regulated proteins, and red for up-regulated proteins, within Cytoscape tool. The exhibited DEPs were arranged by the biological process terms that we selected and those were also presented on PPI network as well.

**Supplemental references**

1. Gillet, L.C., et al., *Targeted Data Extraction of the MS/MS Spectra Generated by Data-independent Acquisition: A New Concept for Consistent and Accurate Proteome Analysis *<sup> </sup>.* Molecular & Cellular Proteomics, 2012. **11**(6).

2. Choi, M., et al., *MSstats: an R package for statistical analysis of quantitative mass spectrometry-based proteomic experiments.* Bioinformatics, 2014. **30**(17): p. 2524-2526.

3. Jiao, X., et al., *DAVID-WS: a stateful web service to facilitate gene/protein list analysis.* Bioinformatics (Oxford, England), 2012. **28**(13): p. 1805-1806.

4. Shannon, P., et al., *Cytoscape: a software environment for integrated models of biomolecular interaction networks.* (1088-9051 (Print)).

5. Szklarczyk, D., et al., *STRING v10: protein-protein interaction networks, integrated over the tree of life.* (1362-4962 (Electronic)).
